# Signature mRNA markers in extracellular vesicles for the accurate diagnosis of colorectal cancer

**DOI:** 10.1186/s13036-020-0225-9

**Published:** 2020-02-04

**Authors:** Byung Seok Cha, Ki Soo Park, Jun Seok Park

**Affiliations:** 1 Department of Biological Engineering, College of Engineering, Konkuk University, Seoul, Republic of Korea; 2 School of Medicine, Kyungpook National University, Daegu, Republic of Korea; 3 Colorectal Cancer Center, Kyungpook National University Chilgok Hospital, Daegu, Republic of Korea

**Keywords:** Colorectal cancer, Extracellular vesicle, mRNA, VEGF, CD133, Non-invasive biomarker

## Abstract

**Background:**

With the increasing incidence of colorectal cancer (CRC), its accurate diagnosis is critical and in high demand. However, conventional methods are not ideal due to invasiveness and low accuracy. Herein, we aimed to identify efficient CRC mRNA markers in a non-invasive manner using CRC-derived extracellular vesicles (EVs). The expression levels of EV mRNAs from cancer cell lines were compared with those of a normal cell line using quantitative polymerase chain reaction. Eight markers were evaluated in plasma EVs from CRC patients and healthy controls. The diagnostic value of each marker, individually or in combination, was then determined using recessive operating characteristics analyses and the Mann-Whitney U test.

**Results:**

Eight mRNA markers (MYC, VEGF, CDX2, CD133, CEA, CK19, EpCAM, and CD24) were found to be more abundant in EVs derived from cancer cell lines compared to control cell lines. A combination of VEGF and CD133 showed the highest sensitivity (100%), specificity (80%), and accuracy (93%) and an area under the curve of 0.96; hence, these markers were deemed to be the CRC signature. Moreover, this signature was found to be highly expressed in CRC-derived EVs compared to healthy controls.

**Conclusions:**

VEGF and CD133 mRNAs comprise a unique CRC signature in EVs that has the potential to act as a novel, non-invasive, and accurate biomarker that would improve the current diagnostic platform for CRC, while also serving to strengthen the value of EV mRNA as diagnostic markers for myriad of diseases.

## Background

Colorectal cancer (CRC) is the second leading cause of cancer-related deaths in males and females and accounts for approximately 10% of all mortalities worldwide. Moreover, according to GLOBOCAN 2018, the Republic of Korea has the third highest cumulative incidence rate of CRC globally, and the highest rate among males [1]. Although a 5-year survival rate of 65% has been applied to CRC, this value drops significantly to 14%, if the cancer metastasizes to other parts of the body [2, 3]. Furthermore, a significantly increased survival rate has been observed in patients with stage I-III compared with those in stage IV, thus, a precise diagnosis within the early stages of disease is extremely critical, as it can contribute to increased survival rates and improved quality of life.

To date, colonoscopic screening and fecal occult blood test (FOBT) have been utilized to diagnose CRC patients in clinical settings [4, 5]. However, these techniques pose serious challenges for accurate diagnosis and effective cancer treatment. The colonoscopic screening is highly invasive and sedation is required, placing a significant burden to the patients. Although the FOBT is non-invasive, it exhibits poor sensitivity with high false positive rates [6–8]. As a promising alternative, liquid biopsy has received special attention, as it allows for non-invasive diagnosis of cancers [9, 10]. The current representative biomarker for CRC diagnosis is carcinoembryonic antigen (CEA) [11]. However, the sensitivity and specificity for CEA detection are fairly poor, making it impractical for screening or diagnosing CRC [7, 12, 13]. In fact, the sensitivities associated with detection of CEA for the diagnosis of CRC are only 4, 25, 44, and 65% in Tumor, Node, Metastasis (TNM) stage I, II, III and IV, respectively [14, 15]. Therefore, new diagnostic markers identified via liquid biopsy with high sensitivity, specificity, and accuracy are needed for improved early diagnosis of CRC, and subsequently improved clinical outcomes.

Small extracellular vesicles (EVs; 50–200 nm), secreted by a myriad of cell types, circulate in the blood and carry genomic and proteomic signatures of their parental cells [16, 17]. In fact, a growing number of studies have demonstrated that EVs function as reliable surrogates of their original cells for non-invasive diagnosis of cancers [18, 19]. Moreover, proteomic analysis of CRC EVs has revealed a number of unique protein markers, including epithelial cell adhesion molecule (EpCAM), cadherin-17, CEA, epidermal growth factor receptor (EGFR), mucin 13 (MUC13), keratin 18, CD147, CD9, and glypican 1 (GPC1) [20, 21]. Additionally, messenger RNAs (mRNAs) have been reported to be differentially expressed between CRC and normal colon tissues; which implies that mRNAs within EVs may serve as potential novel diagnostic biomarkers for CRC diagnosis [22, 23]. However, although studies have reported on microRNAs (miRNAs) within EVs [24–26], the specific mRNAs unique to CRC EVs are not well characterized.

In the current study, we sought to identify reliable biomarkers for CRC diagnosis by selecting putative mRNA biomarkers and evaluating their expression levels within EVs via qPCR in cell lines and clinical samples.

## Results

### Selection of extracellular vesicle mRNA markers

**Fig. 1:**
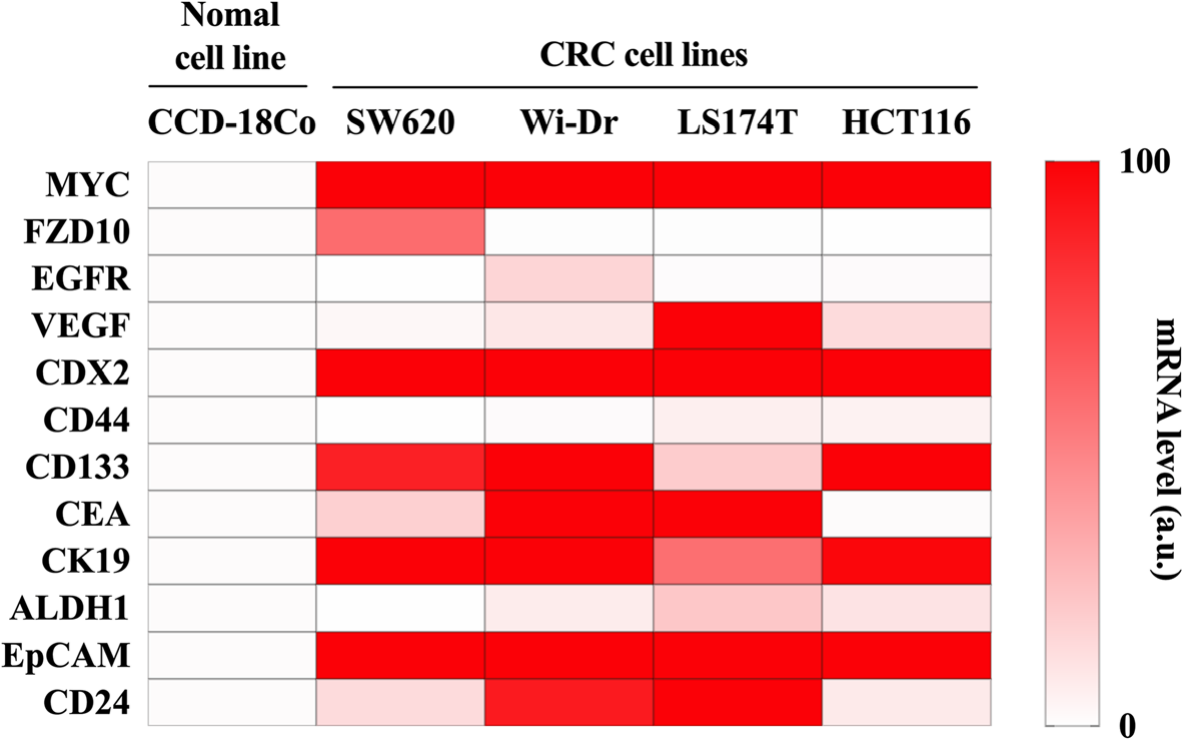
EV mRNA analysis in cell lines. The relative changes in gene expression of each marker from four CRC cell lines (SW620, Wi-Dr, LS174T, and HCT116) were calculated and compared with that in a normal cell line (CCD-18Co) as the control group. EV mRNA markers were selected for further investigation if they were highly expressed in at least one CRC cell line with a relative change in gene expression of ca. 100 (ca, circa); mRNA level (a.u., arbitrary unit) running from bottom to top

To identify appropriate putative mRNA markers for CRC, we searched the available databases (Vesiclepedia, EVpedia, and ExoCarta) and surveyed previous literature of published markers. A total of 12 mRNA markers namely, MYC, Frizzled-10 (FZD10), epidermal growth factor receptor (EGFR), vascular endothelial growth factor (VEGF), caudal type homeobox-2 (CDX2), cluster of differentiation (CD)44, CD133, carcinoembryonic antigen (CEA), cytokeratin-19 (CK19), aldehyde dehydrogenase-1 (ALDH1), epithelial cell adhesion molecule (EpCAM), and CD24, were selected as candidate biomarkers, based on their reported critical roles in CRC pathogenesis (Table 1) [27–35]. To evaluate the mRNA markers for accurate detection of CRC, qPCR was performed after selecting four CRC cell lines (SW620, Wi-Dr, LS174T, and HCT116) and one normal cell line (CCD-18Co). The performance of mRNA markers in differentiating CRC from the control group is summarized in Fig. 1. Based on the heatmap analysis of the 12 EV candidate markers, eight mRNAs (MYC, VEGF, CDX2, CD133, CEA, CK19, EpCAM, and CD24) were determined to be more highly expressed in CRC cell lines compared to the normal cell line and were, therefore, chosen for further analysis.

**Table 1:** Brief description of candidate biomarkers used for CRC diagnosis

No.	Biomarker	Function
1	MYC	• Transcription factor involved in genesis and progression of cancers
2	FZD10	• Transmembrane protein acting as a receptor for the Wingless type MMTV integration site • Upregulated in primary colon cancers
3	EGFR	• Tyrosine kinase receptor that regulates cell growth, differentiation, and angiogenesis
4	VEGF	• Angiogenic factor in CRC • Increased level correlated with advanced lymph node status and distant metastasis
5	CDX2	• Caudal-related homeobox gene that controls cell functions such as adhesion, proliferation, and apoptosis
6	CD44	• Transmembrane glycoprotein that regulates cell adhesion, proliferation, growth, migration, angiogenesis, and differentiation
7	CD133	• Transmembrane glycoprotein identified in colon tumors • High expression associated with distant metastasis, and resistance to chemotherapy and radiotherapy
8	CEA	• Expressed in most cancers • Involved in tumorigenesis by enhancing tumor cell survival and inducing tumor angiogenesis
9	CK19	• Expressed at various levels in epithelial cells • Metastatic once circulated in blood
10	ALDH1	• Important role in early differentiation of cancer stem cells and their proliferation and metastasis
11	EpCAM	• Highly expressed on proliferative, intestinal epithelial cells • Loss is generally associated with a tumor-promoting role
12	CD24	• GPI-anchored membrane protein involved in development and progression of malignant tumors, including CRC

*MYC* myelocytomatosis; *FZD* frizzled; *EGFR* epidermal growth factor receptor; *VEGF* vascular endothelial growth factor; *CDX* caudal type homeobox; *CD* cluster of differentiation; *CEA* carcinoembryonic antigen; *CK* cytokeratin; *ALDH* aldehyde dehydrogenase; *EpCAM* epithelial cell adhesion molecule

### Validation of selected mRNA markers in clinical samples

**Fig. 2:**
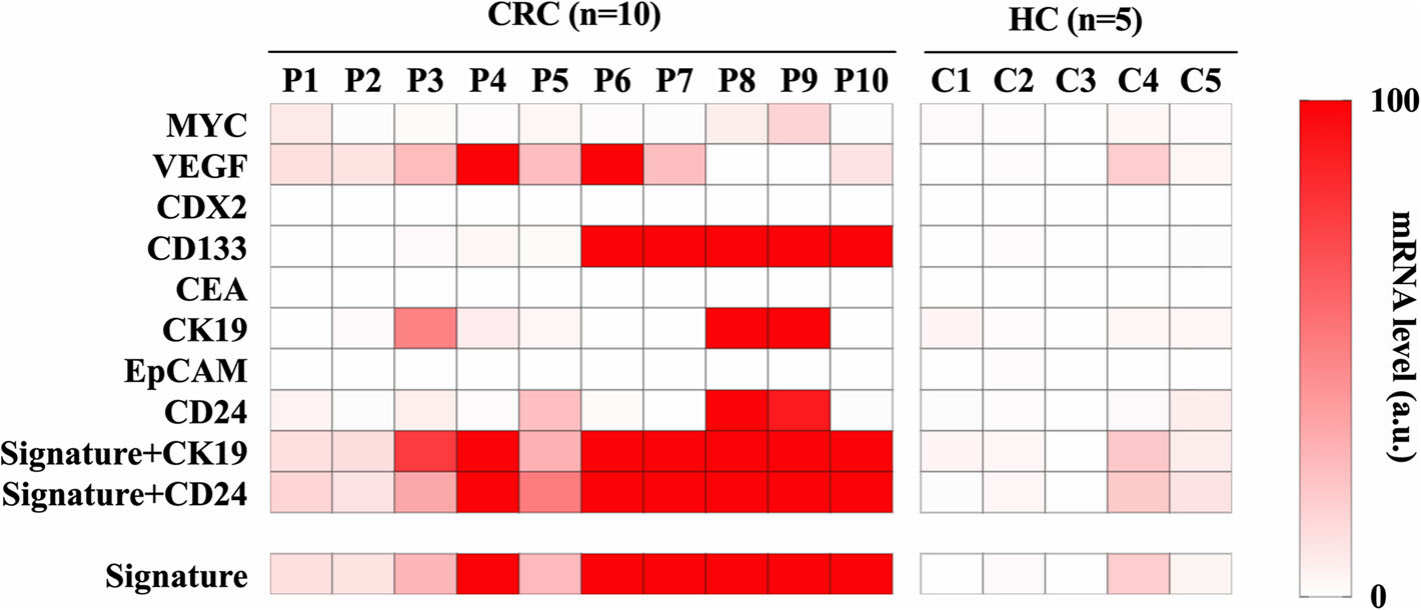
Analysis of clinical samples for expression of specific extracellular vesicle mRNA markers. The relative changes in MYC, VEGF, CDX2, CD133, CEA, CK19, EpCAM and CD24 gene expression from ten CRC patients and five healthy controls were calculated and compared with a healthy control (C2) group (P, CRC patient; HC, Healthy control; C, control; Signature, combined marker of VEGF and CD133); mRNA level (a.u.) running from bottom to top

**Fig. 3:**
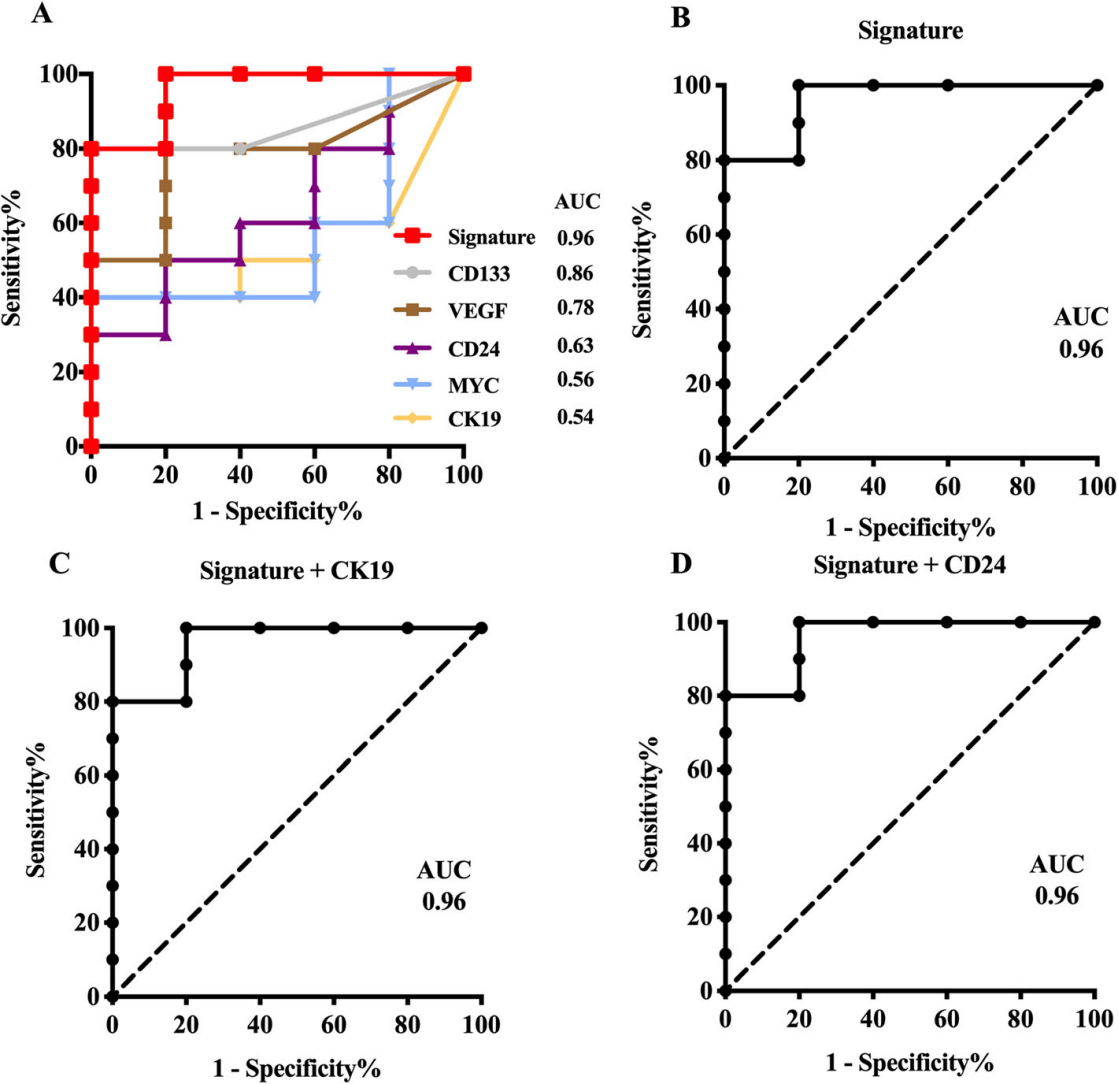
Receiver Operating Characteristic (ROC) curve and Area Under the Curve (AUC). **a** Individual EV mRNA markers (only ROC curves with AUC > 0.5). **b**-**d** Combinations of EV mRNA markers

We next collected plasma from 15 clinical samples consisting of ten CRC patients and five healthy controls (Table 2). The expression levels of eight EV mRNA markers selected from the in vitro experiment (Fig. 1) were evaluated in the plasma samples. After isolation of EVs from the plasma samples, the same procedure as performed in vitro was carried out and the relative change in gene expression of each marker was calculated using healthy participants (C2) as the control group. The heatmap analysis showed that CD133 partially differentiated CRC patients from healthy controls (Fig. 2). However, combining multiple mRNA markers served to improve the ability to differentiate CRC patients from healthy controls. Moreover, the receiver-operating characteristic (ROC) analyses clearly demonstrated that single mRNA markers were unable to meet the requirement for sufficiently high sensitivity, specificity, or accuracy (Fig. 3a). Through a series of comparisons between all possible mRNA combinations, we found that the combining two specific mRNA markers (VEGF and CD133) achieved an area under the curve (AUC) of 0.96 with 100% sensitivity, 80% specificity and 93% accuracy; hence, this was designated as the CRC signature **(**Fig. 3 and Table 3**)**. Importantly, mRNA CEA, the current representative biomarker for CRC diagnosis, was not detectable in both CRC patient and healthy controls, which matches well with the recent report that CEA marker is impractical for screening or diagnosing CRC (Table 3) [36, 37] .

**Table 2:** Demographics of CRC patients employed in the study

Variables	Number of patients
Sex	
M	5
F	5
Age (years)	
< 55	2
55–70	3
> 70	5
Tumor site	
Proximal colon	2
Distal colon	8
Tumor stage (TNM)	
I	0
II	1
III	8
IV	1
Grade of differentiation	
Well	0
Moderate	7
Poor	3

Note: *TNM* tumor-node-metastasis

**Table 3:** Statistical analyses of extracellular vesicle mRNA markers in clinical samples

Biomarker(s)	n	Cut-off^a^	AUC	Sensitivity (%)	Specificity (%)	Accuracy (%)
MYC	1	2.72	0.56	40	100	60
VEGF	1	6.76	0.78	80	80	80
CDX2	1	NA	NA	NA	NA	NA
CD133	1	1.275	0.86	80	100	87
CEA	1	NA	NA	NA	NA	NA
CK19	1	4.85	0.54	40	100	60
EpCAM	1	NA	NA	NA	NA	NA
CD24	1	3.06	0.63	50	80	60
VEGF+CK19	2	8.305	0.94	100	80	93
VEGF+CD24	2	10.19	0.94	100	80	93
CK19 + CD24	2	18.56	0.61	40	100	60
Signature^b^	2	7.19	0.96	100	80	93
Signature+CK19	3	9.49	0.96	100	80	93
Signature+CD24	3	10.52	0.96	100	80	93
Signature+CK19 + CD24	4	38.86	0.91	80	100	80

Note: ^a^ Cut-off value was calculated using Youden’s index, which maximizes the sum of sensitivity and specificity; ^b^ Combined marker of VEGF and CD133; *n* number of biomarker(s); *AUC* area under the curve; *NA* not applicable

**Fig. 4:**
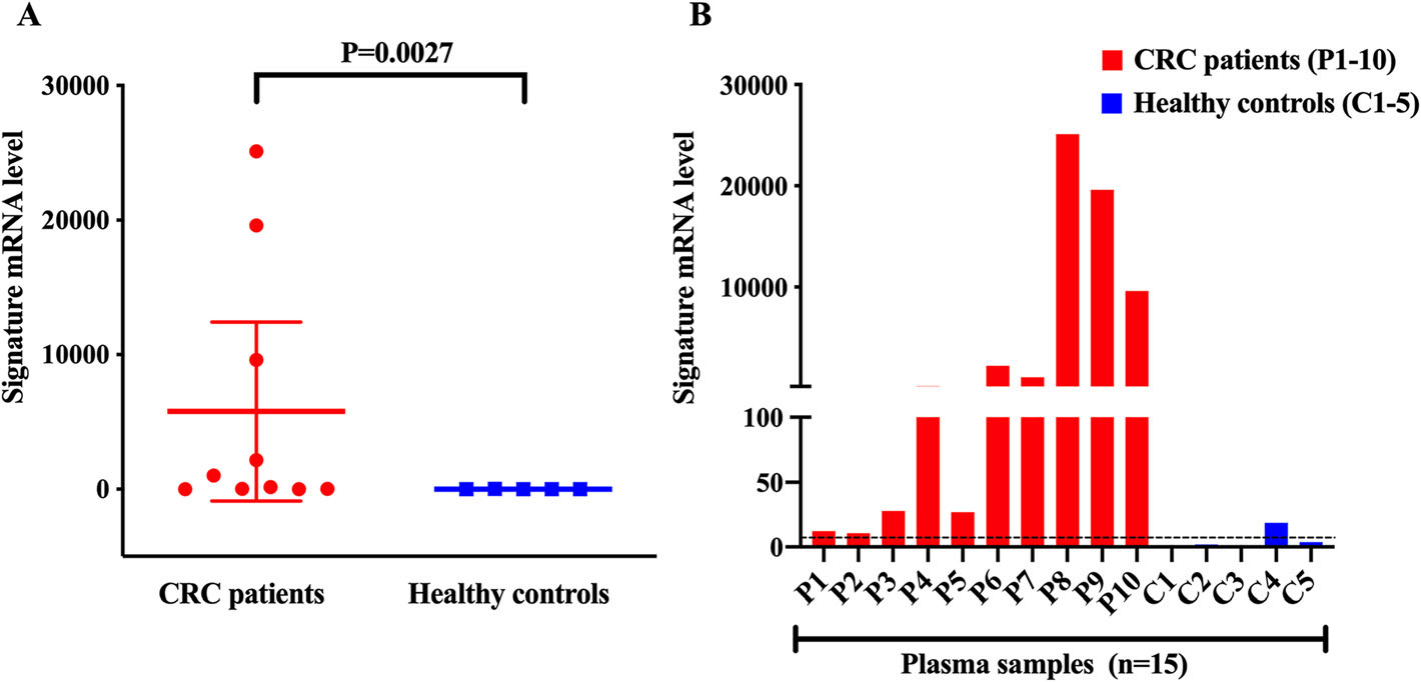
Differentiation of CRC patients from healthy controls using the CRC signature. **a** Relative changes in gene expression of the CRC signature between CRC patients and a healthy control (C2) group. Data are shown as mean + standard deviation. The two-tailed *P* value was determined by Mann-Whitney U test. **b** Bar graph representation for mRNA level of CRC signature in clinical samples. Cut-off value for the CRC signature is shown as a dotted line

Finally, to verify that the CRC signature successfully differentiates CRC patients from healthy controls, the statistical significance of difference was calculated using the Mann-Whitney U test. The results in Fig. 4a show that the expression level of the signature in CRC patients differed significantly from that of healthy controls (*P* = 0.0027). Moreover, the bar graph representation in Fig. 4b indicates that despite one exception that one healthy control (C4) exhibits the higher CRC signature level than cut-off value, the CRC signature level is distinctly higher in the patients compared to the healthy controls, confirming that it has the capacity to serve as a potential CRC biomarker.

## Discussion

EVs have gained increasing attention as diagnostic markers, due to their abundance, prolonged stability and, most importantly, their capacity to non-invasively diagnose different cancers, consequently permitting longitudinal monitoring of patients and reducing the patient’s economic and psychological/emotional burden [21]. A great deal of progress has been made to isolate EVs of high purity from biological fluids and characterize EV biomarkers such as DNA, RNA, and protein. However, scarce attention has been paid to evaluate the diagnostic value of EV mRNA for CRC specifically. Herein, we performed comprehensive analysis using cell lines and patient samples to identify a reliable CRC mRNA marker in EVs that would serve to improve cancer diagnosis and patient management.

On the basis of the hypothesis that EV mRNA levels from cell lines will approximately align with that from clinical samples, four CRC cell lines (SW620, Wi-Dr, LS174T and HCT116) and one normal cell line (CCD-18Co) were selected. Further, 12 mRNA markers were screened to identify eight candidate markers for further validation in clinical samples. From the analysis of the eight candidate markers in clinical samples, no single mRNA marker was found to detect CRC with the desired sensitivity and specificity. Due to the heterogeneous nature of cancer, the expression level of mRNA markers in EVs was variable across individual patients. Therefore, a combination of EV mRNA markers was proposed in anticipation of improved accuracy for the liquid biopsy-based diagnosis. As a consequence, a combination of VEGF and CD133, designated the CRC signature, was found to yield clinically significant values of 0.96 AUC, 100% sensitivity, 80% specificity, and 93% accuracy. These values indicate potential use of the signature as a clinical diagnostic marker for CRC. In fact, triple (CRC signature + CK19 or CD24) and quadruple (CRC signature + CK19 + CD24) markers were also evaluated (Table 3). However, the implementation of triple markers did not significantly improve the detection performance and rather generated identical AUC, sensitivity, specificity, and accuracy values as the duo combinations. Alternatively, in the case of quadruple markers, the AUC, sensitivity, and accuracy values were observed to decrease, whereas specificity increased compared to the duo combinations. Thus, the CRC signature consisting of only the two mRNA markers provided a more robust and cost-effective diagnosis of CRC than the triplet or quadruplet combinations of markers.

There are only a few studies that have investigated mRNA expression in CRC patients. Koga et al. performed experiments with isolated colonocytes from stool and reported that CEA mRNA expression in CRC patients did not differ significantly from that of healthy cohorts (*P* = 0.21, two-sided Mann–Whitney’s U-tests). However, the authors proposed a combination marker composed of matrix metalloproteinase-7 (MMP7), Myb-related protein B (MYBL2), prostaglandin-endoperoxide synthase 2 (PTGS2), and tumor protein 53 (TP53) with 58% sensitivity and 88% specificity [38]. Further, Marshall et al. evaluated the performance of seven combined mRNA markers, namely annexin A3 (ANXA3), C-type lectin domain family 4 member D (CLEC4D), lamin B1 (LMNB1), proline-rich gamma-carboxyglutamic acid protein 4 (PRRG4), tumor necrosis factor alpha induced protein 6 (TNFAIP6), Vanin 1 (VNN1), and interleukin 2 receptor subunit beta (IL2RB) to diagnose CRC patients and achieved 0.80 AUC, 82% sensitivity, 64% specificity, and 73% accuracy [39]. It should be noted that our results performed with EVs displayed higher AUC, sensitivity, and specificity with better accuracy and two-tailed *P*-value (*P* = 0.0027, Fig. 4a), thereby affirming that the CRC signature could efficiently differentiate between CRC patients and healthy controls and thus, may serve as a valuable biomarker for CRC diagnosis.

We believe that this finding improves the CRC diagnostic capacity. Moreover, to the best of our knowledge, this study is the first to conduct an in-depth investigation of EV mRNA markers in both cell lines and CRC clinical samples. Although the results are encouraging, the clinical cohorts were small and thus, further validation of the CRC signature will be required using a large number of clinical samples under various clinical situations: for example, the samples before and after surgery or at different cancer stages. Moreover, the effectiveness of the CRC signature must be examined with other types of cancers to ensure CRC specificity. We believe that these efforts will improve the reliability of the CRC signature, leading to the diagnosis of CRC at an early stage and the reduction of morality rates.

## Conclusions

In summary, the CRC signature composed of VEGF and CD133 mRNAs in EVs was found to be a novel biomarker for the diagnosis of CRC. The data generated in this study may serve as the basis for further investigation and be useful for the development of highly sensitive strategies for rapid and non-invasive monitoring of pathological conditions within CRC patients. Most importantly, in clinical settings where there are no well-established EV mRNA markers, this study is meaningful in that it enables the enhanced diagnosis of CRC and broadens the horizon on the prospective diagnostic capacity of EV mRNA markers.

## Methods

### Reagents and materials

Dynabeads M-270 Epoxy (2.8 μm) and bovine serum albumin (BSA) were purchased from Invitrogen; 1× phosphate-buffered saline (PBS) was ordered from Welgene Inc.; Hyclone™ Dulbecco’s Modified Eagle’s Medium (DMEM), fetal bovine serum (FBS), 100× penicillin-streptomycin solution, and 0.25% (1×) trypsin protease were purchased from GE Healthcare; and exosome-depleted FBS was procured from System Biosciences (SBI). All other reagents were of analytical grade.

### Preparation of immuno-magnetic beads

Immuno-magnetic beads were prepared according to the manufacturer’s protocol. The magnetic beads (5 mg) with epoxy functional groups (Thermo Fisher Scientific) were suspended in 0.1 M sodium phosphate buffer at room temperature for 10 min. The beads were separated from the buffer with a magnetic stand and re-suspended in the same buffer. Based on the optimal reaction ratio [10 μg (antibody):1 mg (bead)] recommended by the manufacturer, a mixture of beads, antibody, and 1 M ammonium sulfate was incubated overnight at 4 °C, with slow tilt rotation. The beads were washed thrice with PBS and re-suspended in PBS with 1% BSA to a final bead concentration of ~ 10^9^ beads/mL. The coupling reaction was allowed to proceed for each antibody (anti-CD9, CD63, and CD81), and all immuno-magnetic beads were combined to enhance EV-capturing efficiency.

### Cell culture

All cell lines used in this study were obtained from the Korean Cell Line Bank. The human normal colon cell line CCD-18Co [40, 41] as well as the human colon cancer cell lines SW620, Wi-Dr, LS174T, and HCT116 were cultured in DMEM supplemented with 10% (v/v) FBS, 100 U/mL penicillin, and 100 μg/mL streptomycin at 37 °C in a humidified atmosphere of 5% CO_2_. Approximately 10^6^ cells at passage number 1–15 were cultured in 150-mm culture dish until ~ 80% cellular confluence was observed.

### Extracellular vesicle isolation from in vitro cultured cells

All cell lines showing ~ 80% cellular confluence were cultured in conditioned media supplemented with 5% (v/v) vesicle-depleted FBS for 48 h at 37 °C in a humidified atmosphere of 5% CO_2_. EVs were isolated from the conditioned medium using a conventional method [42]. Briefly, the conditioned medium was collected in a sterile tube and centrifuged at 300×*g* for 5 min to remove suspended cells. The supernatant was then filtered through a 0.2-μm cellulose acetate membrane filter (Corning, 431,219) and ultra-centrifuged at 4 °C for 1 h at 100,000×*g* to pellet EVs. After discarding the supernatant, the EV pellet was washed with PBS once and centrifuged at 100,000×*g* for 1 h. After aspiration of the PBS supernatant, the EV pellet was re-suspended in PBS and stored at − 80 °C until use.

### Clinical samples

A total of ten CRC patients and five healthy individuals were registered from the Colorectal Cancer Clinics at Kyungpook National University Chilgok Hospital (KNUCH) between January 2017 and October 2018 (Table 2). An equal number of males and females were enrolled, with the age ranging from 50 to 83 years, and a mean age of 68.6 years. Of the ten CRC patients, one was in TNM stage II, eight were in TNM stage III, and one was in TNM stage IV. For clinical sample acquisition, peripheral blood (~ 15 mL) was withdrawn from the patients and healthy volunteers (normal controls). Peripheral blood samples were collected in ethylenediaminetetraacetic acid (EDTA) tube by the hospital staff and immediately centrifuged at 1500×*g* for 10 min at 4 °C. The resulting supernatant, designated as the serum, was carefully collected and stored at − 80 °C until use.

The clinical research protocol was approved by the Institutional Review Board (IRB) at KNUCH. After providing detailed explanation, informed written consent was obtained from all patients and healthy volunteers according to the IRB-approved clinical research protocol. CRC was medically confirmed in eligible patients aged < 80 years by a colonoscopic biopsy. For evaluation of distant metastasis, an abdominopelvic and a chest computed tomography (CT) were performed. For the purpose of this study, we thoroughly checked healthy individuals for history of other malignancies and their records of comprehensive medical examination in the past one year. The participants were recruited from the public through posters displayed at KNUCH. We believe our samples are representative of a large population, although a larger scale study is warranted to confirm our results.

### EV isolation from clinical samples

Human serum EVs were isolated using immuno-magnetic beads conjugated with combined antibodies [43]. Specifically, each designated human serum was first added to a prefabricated mixture of immuno-magnetic beads with anti-CD9, CD63, and CD81 antibodies and incubated overnight at 4 °C, with slow tilt rotation. Next, the whole solution was placed on a magnetic stand and the supernatant was carefully removed without disturbing the magnetic beads. Immuno-magnetic beads were then washed thrice with PBS and re-suspended in PBS and used immediately for further experiments.

### Extracellular vesicle RNA extraction

EV samples isolated from cell culture media and plasma were mixed with TRIzol reagent (Thermo Fisher Scientific) and total RNA from the EVs was extracted using Direct-zol RNA kit (Zymo research), according to manufacturer’s protocol. The concentration and quality of extracted RNA were determined using Nanodrop spectrophotometer (Thermo Fisher Scientific) and 2100 Bioanalyzer (Agilent) using a RNA 6000 Pico Chip. RNA samples with RNA integrity number (RIN) above 9 were used for further analysis (RIN 1 to 10 indicates highly degraded to completely intact, respectively).

### mRNA analysis

Approximately 100 ng of extracted EV RNAs were reverse-transcribed to generate cDNA using a high-capacity RNA-to-cDNA kit (Thermo Fisher Scientific), following the manufacturer’s protocol, and were pre-amplified in the case of patient samples using Taqman PreAmp Master Mix (Thermo Fisher Scientific), prior to the quantitative polymerase chain reaction (qPCR) experiments. All reactions were performed using Taqman Gene Expression Master Mix and Taqman Gene Expression Assays (Thermo Fisher Scientific) on an ABI 7500 Fast Real-Time PCR system (Applied Biosystems), as recommended by the manufacturer. Amplification for qPCR experiments was performed with the following conditions: 50 °C for 2 min, 95 °C for 10 min, followed by 40 cycles of 95 °C for 15 s and 60 °C for 1 min. Primers for each biomarker are listed in Additional file 1 Table S1 and were purchased from Thermo Fisher Scientific. All experiments were carried out in triplicate. The relative quantification was calculated by the 2^-ΔΔCt^ method and normalized to the respective GAPDH expression and the linear combination of markers was calculated as the following equation: $y={\sum}_{i=1}^n{x}_i$ where y is total expression level of combined markers, x is individual expression level of a marker, and i and n represent the first and the last term of combined markers, respectively.

### Statistical analysis

Mann-Whitney U tests were performed to determine the statistical significance in the differences between EV mRNAs from CRC and healthy controls. ROC curves were established, and AUC was calculated to evaluate the performance of selected EV mRNAs as a diagnostic marker for CRC. All statistical analyses were performed using GraphPad Prism 7 software (GraphPad software, Inc., La Jolla, CA, USA). A *P*-value < 0.05 was considered to be statistically significant.

## Supplementary information

**Additional file 1: Table S1.** Primers used in qPCR for extracellular vesicle mRNA analyses.

## Data Availability

All data generated or analyzed during this study are included in this published article and its additional files.

## Abbreviations

ALDH1: Aldehyde dehydrogenase 1
ANXA3: Annexin A3
AUC: Area under the curve
BSA: Bovine serum albumin
C: Control
CD: Cluster of differentiation
CDX: Caudal type homeobox
CEA: Carcinoembryonic antigen
CEACAM: Carcinoembryonic antigen-related cell adhesion molecule
CK: Cytokeratin
CLEC4D: C-type lectin domain family 4 member D
CRC: Colorectal cancer
DMEM: Dulbecco’s Modified Eagle Medium
EDTA: Ethylenediaminetetraacetic acid
EGFR: Epidermal growth factor receptor
EpCAM: Epithelial cell adhesion molecule
FBS: Fetal bovine serum
FOBT: Fecal occult blood test
FZD10: Frizzled-10
GAPDH: Glyceraldehyde 3-phosphate dehydrogenase
GPC1: Glypican 1
HC: Healthy control
IL2RB: Interleukin 2 receptor subunit beta
KRT: Keratin
LMNB1: Lamin B1
miRNAs: microRNAs
MMP7: Matrix metalloproteinase-7
Muc13: Mucin 13
MYBL2: Myb-related protein B
MYC: Myelocytomatosis
P: CRC patient
PBS: Phosphate buffered saline
PRRG4: Proline-rich gamma-carboxyglutamic acid protein 4
PTGS2: Prostaglandin-endoperoxide synthase 2
ROC: Receiver-operating characteristic
TNFAIP6: Tumor necrosis factor alpha induced protein 6
TNM: Tumor, Node, Metastasis
TP53: Tumor protein 53
VEGF: Vascular endothelial growth factor
VNN1: Vanin 1
