# Supplementary material for: Signature mRNA markers in extracellular vesicles for the accurate diagnosis of colorectal cancer
Source: J Biol Eng. 2020 Feb 4;14:4. doi: 10.1186/s13036-020-0225-9 (PMC7001337; doi:10.1186/s13036-020-0225-9)
Supplement: Supplementary file 1 — Additional file 1: Table S1. Primers used in qPCR for extracellular vesicle mRNA analyses. [file 13036_2020_225_MOESM1_ESM.doc]

**Table S1 Primers used in qPCR for extracellular vesicle mRNA analyses**

| Biomarker | Taqman | |
| --- | --- | --- |
|  | ID | Product Size (bp) |
| MYC | Hs00153408_m1 | 107 |
| FZD10 | Hs00273077_s1 | 116 |
| EGFR | Hs01076090_m1 | 57 |
| EpCAM | Hs00901885_m1 | 95 |
| CD24 | Hs02379687_s1 | 140 |
| CD44 | Hs01075864_m1 | 79 |
| CD133 | Hs01009259_m1 | 66 |
| CEA (CEACAM5) | Hs00944025_m1 | 71 |
| CK19 (KRT19) | Hs00761767_s1 | 116 |
| ALDH1 (ALDH1A1) | Hs00946916_m1 | 61 |
| VEGF (VEGFA) | Hs00900055_m1 | 59 |
| CDX2 | Hs01078080_m1 | 81 |
| GAPDH | Hs03929097_g1 | 58 |

MYC, myelocytomatosis; FZD10, frizzled-10; EGFR, epidermal growth factor receptor; EpCAM, epithelial cell adhesion molecule; CD, cluster of differentiation; CEA, carcinoembryonic antigen; CEACAM, carcinoembryonic antigen-related cell adhesion molecule; CK, cytokeratin; KRT, keratin; ALDH, aldehyde dehydrogenase; VEGF, vascular endothelial growth factor; CDX, caudal type homeobox; GAPDH, Glyceraldehyde 3-phosphate dehydrogenase
